# Resveratrol Interferes with IL1-β-Induced Pro-Inflammatory Paracrine Interaction between Primary Chondrocytes and Macrophages

**DOI:** 10.3390/nu8050280

**Published:** 2016-05-11

**Authors:** Emeric Limagne, Allan Lançon, Dominique Delmas, Mustapha Cherkaoui-Malki, Norbert Latruffe

**Affiliations:** 1 Laboratoire de Lipides, Nutrition, Cancer, Université de Bourgogne-Franche Comté, Dijon F21000, France; limagne.emeric@yahoo.fr (E.L.); allan.lancon@gmail.com (A.L.); dominique.delmas@u-bourgogne.fr (D.D.); malki@u-bourgogne.fr (M.C.-M.); 2 Laboratoire de Biochimie du Peroxysome, Inflammation et Métabolisme des Lipides (BioPeroxIL EA 7270), Faculté des Sciences Gabriel, Dijon F21000, France; 3 “Chemotherapy, Lipid Metabolism and Antitumoral Immune Response” Team, Faculty of Health Sciences, INSERM (Institut National de la Santé et de la recherché Médicale) Research Center U866, Dijon F21000, France

**Keywords:** IL1-β, chondrocyte, macrophage, NF-κB, STAT3, resveratrol

## Abstract

*State of the art*. Osteoarthritis (OA) is a chronic articular disease characterized by cartilage degradation and osteophyte formation. OA physiopathology is multifactorial and involves mechanical and hereditary factors. So far, there is neither preventive medicine to delay cartilage breakdown nor curative treatment. *Objectives.* To investigate pro-inflammatory paracrine interactions between human primary chondrocytes and macrophages following interleukin-1-β (IL-1β) treatment; to evaluate the molecular mechanism responsible for the inhibitory effect of resveratrol. *Results*. The activation of NF-κB in chondrocytes by IL-1β induced IL-6 secretion. The latter will then activate STAT3 protein in macrophages. Moreover, STAT3 was able to positively regulate IL-6 secretion, as confirmed by the doubling level of IL-6 in the coculture compared to macrophage monoculture. These experiments confirm the usefulness of the coculture model in the inflammatory arthritis-linked process as a closer biological situation to the synovial joint than separated chondrocytes and macrophages. Il also demonstrated the presence of an inflammatory amplification loop induced by IL-1β. Resveratrol showed a strong inhibitory effect on the pro-inflammatory marker secretion. The decrease of IL-6 secretion is dependent on the NFκB inhibition in the chondrocytes. Such reduction of the IL-6 level can limit STAT3 activation in the macrophages, leading to the interruption of the inflammatory amplification loop. *Conclusion.* These results increase our understanding of the anti-inflammatory actions of resveratrol and open new potential approaches to prevent and treat osteoarthritis.

## 1. Introduction

### 1.1. Osteoarthritis

Arthrosis or osteoarthritis (OA) is a chronic disease that mainly affects joints. It appears as intense pain caused by an unusual attrition of joint cartilage and of the linked bones. OA is an age-linked pathology that handicaps senior people. Increasing life expectancy classifies this disease among true public health concerns. Although the osteoarthritis does not represent a major cause of mortality, this pathology is painful and incapacitating. 

Decreased biomechanical and biological capacities of the joint result in an unbalance between the anabolism and catabolism of cartilage matrix proteins (*i.e.*, type II collagen and fibronectin). These proteins are produced by chondrocytes, which represent the unique cell type of cartilage. Chondrocytes express the entire genetic repertoire required to synthesize the different matrix proteins (anabolism) while secreting metalloproteinases that breakdown collagen to allow its renewal catabolism [1]. During the process of aging, the decrease of matrix proteins synthesis favors hydric loss in the cartilage. This process is also characterized by a strong production of proteoglycans, the synthesis of type I collagen and chondrocyte activation. This allows the secretion in the synovial liquid of proteases, cytokines and pro-inflammatory mediators, including Interleukin-1β, PGE2 and TNF-α [2]. These molecules stimulate chondrocytes apoptosis, inhibit the synthesis of cartilage basic components and facilitate attraction and activation of immune cells (monocytes, neutrophils) as well as the activation of synoviocytes, the cells of the synovial membrane. This inflammatory environment leads to the breakdown of the cartilage and uncovers the sub-chondral bone, allowing the formation of osteophytes (bone overgrowth) and increasing bone mechanical frictions in the joint.

Currently there are neither preventive therapeutics to delay cartilage breakdown nor curative treatment. Nevertheless, some medications are able to release pain and joint rigidity. Beside healthy recommendations, such as loss of weight, moderated physical activity and appropriated diet, meanwhile, during the acute inflammatory phase, the use of oral analgesics, *i.e.*, paracetamol and non-steroid anti-inflammatory drugs (NSAID), *i.e.*, rofecoxib, celecoxib, as inhibitors of cyclo-oxygenase 2, or steroid anti-inflammatory drugs (SAID), *i.e.*, cortisone, dexamethasone, is unavoidable. These treatments are somewhat time-limited due to luckless secondary effects (strong hepatic and digestive toxicities). There are some alternates to oral anti-inflammatory drugs such as topical applications to the painful joint (Voltaren^®^) or infiltrations of corticosteroids or hyaluronic acid. Other medications such as glucosamine or chondroitin (basic components of cartilage) supplementation can be also prescribed [3]. In case of the failure of these above-mentioned treatments, surgery can be undertaken through the use of arthroscopy, which allows the removal of cartilaginous or bone debris. Despite this arsenal of treatments, the efficiency of treatment remains still limited. This is why more and more studies are now focusing on the use of herbal medicine or natural molecules present in food (functional food), which exhibit pain attenuation and anti-inflammatory properties [4]. Among them is resveratrol (3,4′,5-trihydroxy-*trans-stilbene*), a well-known dietary polyphenol of the stilbene family, which is considered able to decrease inflammatory processes [5].

### 1.2. Resveratrol and Joint Inflammation

At the cellular level, resveratrol can inhibit several signaling pathways involved in inflammation and in biological processes leading to the attenuation of pain [6,7]. In a rabbit induced inflammatory arthritis model induced by LPS (Lipo Poly Saccharide) injection in the knee, the infiltration of resveratrol (2 mg/kg body weight/day) in the joint allows the limitation of the pro-inflammatory effects of LPS and the prevention of cartilage breakdown [8]. Another study on a surgically induced osteoarthritis model shows that the administration of resveratrol (10 mg/kg body weight/day) limits cartilage breakdown, chondrocyte apoptosis and nitrogen monoxide production in the synovial liquid [9]. *In vitro*, the stimulation of primary chondrocytes by IL-1β or by TNF-α leads to overexpression of enzymes such as matrix metalloproteinases (MMPs) [10], cyclooxygenases (COX1 and COX2) and the secretion of pro-inflammatory cytokines such as IL-6 [11], IL-8 and IL-1β [12], and TNFα due to the activation of NF-κB transcription factor [13]. Interestingly, resveratrol/IL-1β co-treatment decreased NF-κB activation [14] and largely inhibited the activation of pro-inflammatory genes. In addition, resveratrol is a direct inhibitor of cyclooxygenase 2 (COX2) which produces pro-inflammatory lipid mediators responsible for pain sensation (leukotrienes and prostaglandins) [15]. Collectively, the above data clearly establish resveratrol’s anti-inflammatory capacities, based on its ability to target key factors of inflammation such as COX2 and NF-κB [16]. Furthermore, Shakibaei *et al.* [17] reported that resveratrol inhibits IL-1β-induced stimulation of caspase-3 and cleavage of PARP in human articular chondrocytes *in vitro*. Csaki *et al.* [18] were the first to study the regulation of inflammatory signals by resveratrol in human chondrocytes *in vitro*. Häusler *et al.* [19] reported that osteoclast formation requires the presence of macrophage colony-stimulating factor In this way, we have studied the potential effect of resveratrol as an anti-inflammatory compound to counteract the production of pro-inflammatory cytokines such as IL-6, IL-8 in a coculture chondrocyte/macrophage model. Resveratrol’s ability to fight inflammation in this model could provide a new strategy for the treatment of osteoarthritis.

## 2. Methods

### 2.1. Coculture Model

**Figure 1:**
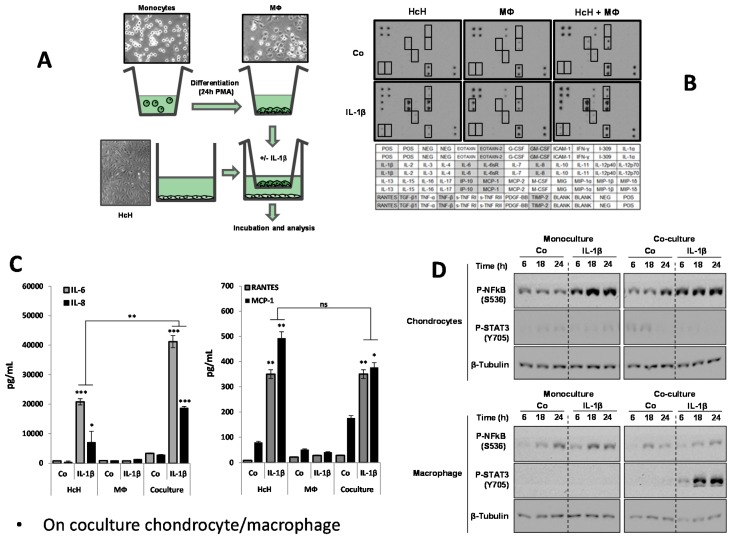
Characterization of inflammation in chondrocyte/macrophage coculture model. (**A**) Scheme of coculture model device and morphology of the grown cells as seen by contrast phase optical microscopy. HcH: Human chondrocytes. MΦ: Macrophages; (**B**) Representation of antibody-array screening of cytokines, 24 h after IL-1β treatment (IL-1β) or not (Co) on primary human chondrocytes (HcH) alone, Thp1-derived macrophages (MΦ) alone or in our coculture system with both HcH and MΦ. Table above shows the antibody array representing anti-cytokines antibody emplacement; (**C**) ELISA determination of the levels of IL-6, IL-8, Rantes, MCP-1 in HcH, MΦ cultures or in HcH/MΦ coculture. Effect of IL-1β treatment. Pg/mL: picogram/mL. *, *p*-value ≤ 0.05; **, *p*-value ≤ 0.01; ***, *p*-value ≤ 0.001. Statistical analysis: data are the means ± SEM of three different experiments and assays in triplicate. Differences between groups were analyzed by one-way analysis of variance (ANOVA); (**D**) Western blot assessment of the level of phosphorylated NFκB (p-NFκB) and p-STAT3 in chondrocytes and in macrophages. Influence of the monocultures and coculture conditions. Β-Tubulin was used as the control of protein loading. Concerning STAT3 phosphorylated changes, we assumed the level of un-phosphorylated STAT3 was unchanged since we have seen no changes in its level in Figure 2B and Figure 5B. For NF-κB, we titrated the amount of phospho-NF-κB with a specific phospho antibody, but so far we have no indication on the molecular mechanism of interconversion between unphosphorylated and phosphorylated forms.

In order to simulate the intercellular interactions, which take place in an arthritic joint, we used the coculture system of BD Bioscience Co., Franklin Lakes, NJ, USA. This device allows the growth of two cell types in two separated wells, and their re-assembly when needed (Figure 1A). Human primary chondrocytes and macrophages were stimulated by IL-1β. This interleukin was chosen due to its key role in the osteoarthritis pathogenesis, after validation of its pro-inflammatory effect on human chondrocytes, compared to those of TNFα. Promo Cell Co. provided human primary chondrocytes isolated from the cartilage of healthy humans having cartilaginous resection (orthopedic surgeon). The quality of the tissues was evaluated by cell morphology analysis (star form), by testing the expression two chondrocyte markers (collagen II and proteoglycan) as well as the capacity of chondrocytes to produce cartilage when grown in semi-liquid medium. After thawing, frozen chondrocytes can be maintained in a differentiated culture up to two weeks. However, to avoid any possible chondrocyte de-differentiation, as soon as the cell density was appropriated (usually before two weeks), the protocols for testing the inflammatory markers were launched. Moreover, we maintained the treatment of cultured cells no longer than 24 h.

Macrophages were obtained by inducing the *in vitro* differentiation of monocytes Thp-1 human monocytes for 24 h treatment by PMA (Phorbol-myristate acetate, 50 nM). Monocyte differentiation was evaluated from their morphological changes. Monocytes have a spherical shape and do not attach to the flask plastic support, while macrophages have a fibroblastic shape and stick to the support. These macrophages express typical makers such as CD11b and show the capacity to respond to inflammatory stimuli (lipopolysaccharides, cytokines).

Chondrocytes were grown in one well, while monocyte differentiation was induced in a cupule whose porous bottom (0.4 μm pore) allows the free diffusion of soluble factors present in the culture medium. Following macrophage differentiation, the two cell types (macrophages and chondrocytes) were assembled in the same culture medium, thus allowing us to simultaneously stimulate the two cell types with IL-1β (2 ng/mL). Cells were then incubated at 37 °C for different time periods (6, 18 or 24 h). As a control, the protocol was conducted on the two cell types separately.

### 2.2. Markers Estimation

Primary human chondrocytes and macrophages were cocultured with or without IL-1β. The expression of pro-inflammatory markers was identified and measured respectively by cytokine antibody array and by ELISA. NF-κB and STAT3 as signaling elements involved in cytokine production were analyzed by Western blotting both in chondrocytes and in macrophages. Chemical inhibitors were used to confirm the importance of these pathways. The crucial role of IL-6 in our model was evaluated by using anti-IL-6 (from Merck Chimie SAS 201, Rue Carnot, Fontenay sous Bois, Île-de-France 94126, France), p65 antibody S536 (anti p-NF-κB) and Y705 (anti p-STAT3 antibody) (from Abcam, 24 rue Louis Blanc, 75010 PARIS, France), the NF-κB phosphorylation inhibitors (BAY-11-7082) (from Merck Chimie SAS, 201, Rue Carnot, Fontenay sous Bois, Île-de-France 94126, France), and STA21 (STAT3 phosphorylation inhibitor) (from Medchemexpress Europe Box 128-521-1298 Stockholm, Sweden).

## 3. Results

### 3.1. Response of Chondrocytes to IL-1β

IL-1β is an interleukin largely involved in the arthritic process. Its pro-inflammatory effects on primary chondrocytes were evaluated towards primary chondrocytes following 24 h of IL-1β treatment (2 ng/mL). Figure 1B reports the cytokine antibody-array screening in chondrocytes, in macrophages or in the coculture medium. These results show that IL-1β-treated chondrocytes secrete the following cytokines or their related receptors: Rantes, TGF-β, TNF-β, IL-6, IL-6sR, IL-8, IP-10, MCP-1, TIMP-2. No effect of IL-1β was observed in macrophages. In cocultures, the effects of IL-1β remained the same as in chondrocyte monoculture. The levels of expression of IL-6, IL-8, MCP-1 and Rantes are presented in Figure 1C. It shows a synergistic effect of macrophages in coculture for IL-6 and IL-8, with a significant decrease for MCP-1 and Rantes. On the other hand, the expression of key signaling factors, the NF-κB and STAT-3 pathways, in their phosphorylated form was cell-type specific. Indeed, Figure 1D shows that the level of phosphorylated NF-κB in chondrocytes was enhanced in the IL-1β-treated monoculture, while no changes were seen in the IL-1β-treated coculture. The level of phosphorylated STAT3 in chondrocytes remains low regardless of the conditions of the culture and treatment. In coculture only the level of phosphorylated STAT3 in macrophages is strongly increased, followed by IL-1β-treatment. No significant changes of were observed in the level of phosphorylated NF-κB in macrophages after IL-1β treatment in monoculture as well as in coculture.

### 3.2. Characterization of Coculture Model Chondrocyte/Macrophage

In coculture conditions, IL-1β also induced morphological changes of chondrocytes but this effect was stronger than in the absence of macrophages (Figure 1C). This indicates a cross-talk between the two cell types where cytokines are differently expressed. It is possible that IL-1 β might increase pro-inflammatory cytokine secretion in chondrocytes, which would then target macrophages and create an amplification loop. IL-1β induced the production of IL-6 by chondrocytes, a potent inflammatory cytokine in chondrocytes, reaching a peak at 18 h (not shown). Interestingly (Figure 1C), IL-1β induced a high level of cytokine production (IL-6, IL-8) in the coculture when compared to the monocultures, while it is the opposite for Rantes and MCP-1. In contrast, macrophages did not produce these cytokines (Figure 1C). These findings suggest that the inflammation mechanism process might implicate two signaling pathways. NFκB transcription factor plays a major role in the inflammatory response by increasing the transcription of genes encoding pro-inflammatory cytokines, especially IL-6. As expected, NF-κB phosphorylation was increased in IL-1β-treated chondrocytes, both in monoculture and in coculture conditions, while no significant activation was seen in macrophages (Figure 1D). This indicates that NF-κB does not play a key role in the inflammatory amplification loop. Therefore, the activation of STAT3, another nuclear factor involved in the inflammatory process, was subsequently explored. STAT3 can also be activated by numerous cytokines and especially IL-6. Indeed, STAT3 factor was phosphorylated only after treatment with IL-6 containing supernatant with IL-1β-treated chondrocytes (Figure 1D).

**Figure 2:**
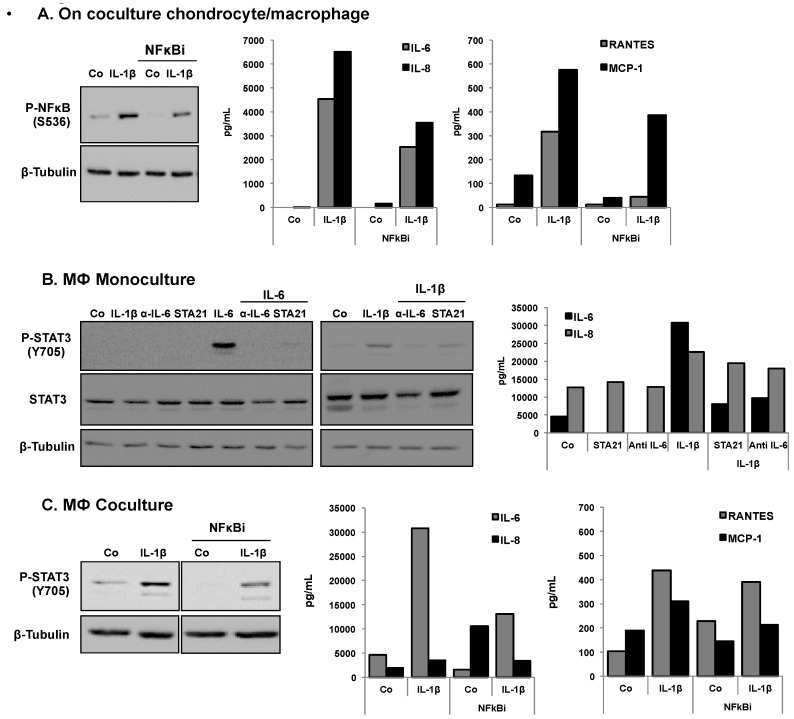
Effects of a NF-κB inhibitor on the levels of p-NF-κB and p-STAT3 and on the levels of secreted IL-6, IL-8, Rantes and MCP-1 in HcH, MΦ cultures or in HcH/MΦ coculture. (**A**) Effect of a NF-κB inhibitor on the level of p-NF-κB (Western blot) and on the levels (ELISA) of IL-6, IL-8, Rantes, MCP-1 in HcH cultures and the effect of IL-1β treatment. Representative data from one of three experiments are shown; (**B**) Levels of p-STAT3 were determined by Western blotting) and the levels of IL-6 and IL-8 were determined by ELISA assay, in MΦ cultures. Effect of treatment with IL-1β, IL-6, aIL-6 or STA21. Co, control. Representative data from one of three experiments are shown; (**C**) Expression of p-STAT3 (Western blotting) and on the level (ELISA) of IL-6, IL-8, Rantes, MCP-1 in MΦ cocultures. Effect of IL-1β and NF-κB inhibitor. β-Tubulin: protein loading control, Pg/mL: picogram/mL. Representative data from one of three experiments are shown.

This strongly suggests that paracrine interactions between chondrocytes and macrophages were taking place. To confirm this hypothesis, we tested the effect of different inhibitors of phosphorylation of p-NF-κB and p-STAT3, in addition to the measurement of secretion levels of IL-6, IL-8, Rantes and MCP-1 (Figure 2). Indeed, the NF-κB inhibitor (NF-κBi) decreased both p-NF-κB and cytokine levels following IL-1β stimulation (Figure 2A). Figure 2B shows a decrease of the p-STAT3 level and of IL-6, and of IL-8 production in IL-1β-stimulated macrophages in monoculture treated with STA21 (STAT3 inhibitor), anti-IL-6 antibody and alpha IL-6. Figure 2C indicates that for macrophages in coculture, the level of p-STAT3 decreased in the presence of the NF-κB inhibitor (NF-κBi) coculture. Accordingly, there was also a decrease of the levels of IL-6, IL-8, Rantes and MCP-1.

### 3.3. Effects of Resveratrol

**Figure 3:**
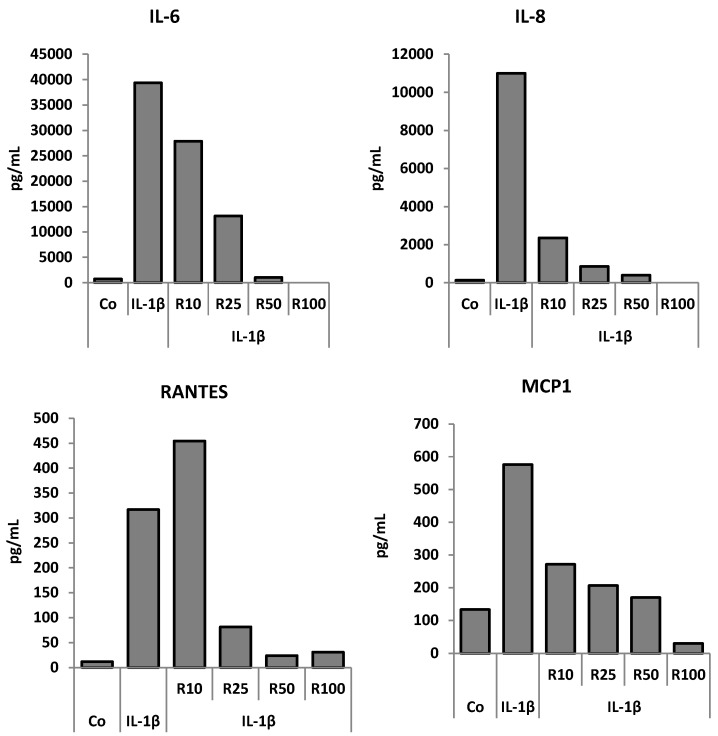
Resveratrol effects on the levels of secreted IL-6, IL-8, Rantes and MCP-1 in HcH monoculture and influence of the IL-1β treatment. Representative data from one of three experiments are shown. ELISA assessment of the levels of IL-6, IL-8, Rantes, MCP-1 in culture medium. Co: control. Resveratrol (R) in μM. Pg/mL: picogram/mL.

**Figure 4:**
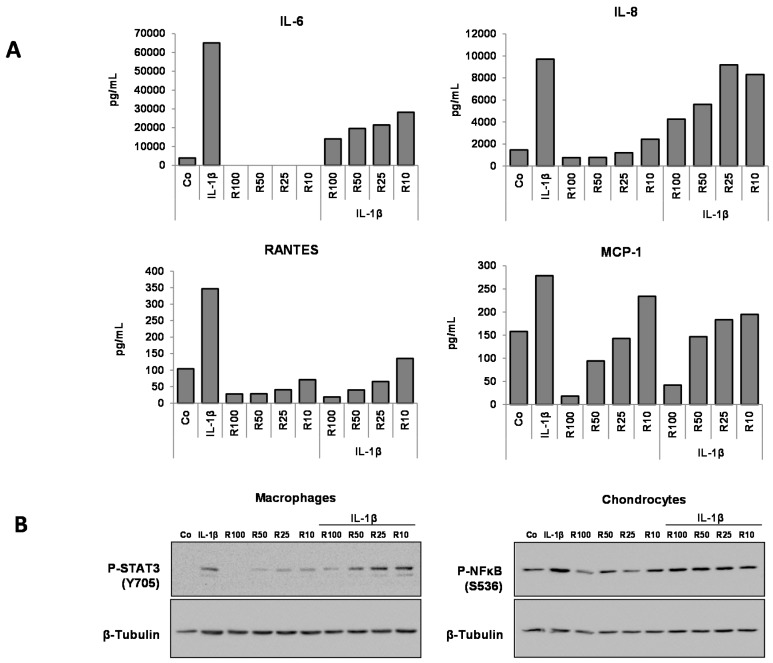
Resveratrol effect on the expression levels of IL-6, IL-8, Rantes, MCP-1 and on the expression of p-NF-κB and p-STAT3 in HcH and MΦ cocultures and influence of the IL-1β treatment. (**A**) ELISA estimation of the level of IL-6, IL-8, Rantes, MCP-1 in culture medium. Representative data from one of three experiments are shown; (**B**) Western blotting assessment of the expression of p-STAT3 in HcH and MΦ cocultures. β-Tubulin: protein loading control, Co: control. Resveratrol (R) in μM. Pg/mL: picogram/mL. Representative data from one of three experiments are shown.

**Figure 5:**
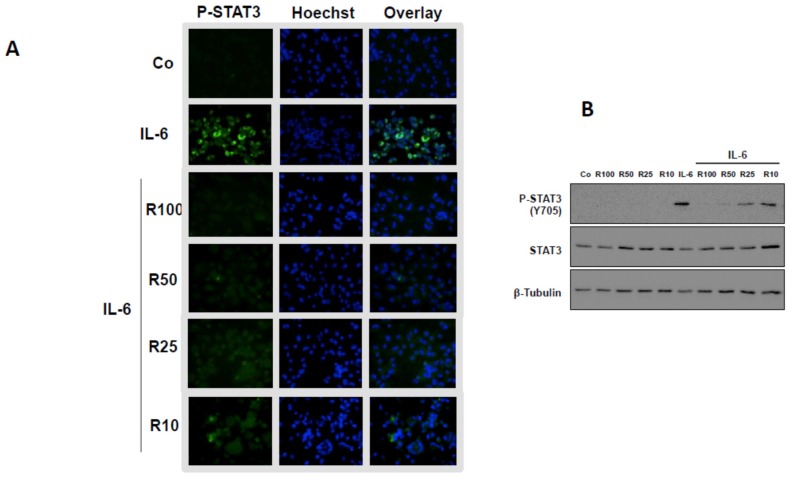
Resveratrol effects on the levels of STAT3 phosphorylation in macrophage monoculture. (**A**) Fluorescence microscopy analysis of STAT3 phosphorylation (green labeling). Resveratrol (100 or 50, 25 or 10 μM) limits the IL-6-induced STAT3 phosphorylation level (decrease of green labeling). Cell nuclei were labeled with Hoechst dye. The overlay confirms the nuclear location of p-STAT3. Representative data from one of three experiments are shown; (**B**) Western blotting analysis of STAT3 phospholylation. Resveratrol limits the IL-1β-induced STAT3 phospholylation. β-Tubulin: protein loading control; Co, control. Representative data from one of three experiments are shown.

Results reported in Figure 3 show that resveratrol was able to diminish the levels of secreted IL-6 as well as those of IL-8, Rantes, and MCP-1 in the monoculture of chondrocytes and macrophages. Resveratrol decreased the IL-6 level as well as those of IL-8, Rantes, and MCP-1 in a dose-dependent manner (Figure 4A). This is noteworthy, given that resveratrol strongly limited STAT3 activation in macrophages but exhibited no effect towards NF-κB’s phosphorylated form in chondrocytes (Figure 4B). The decreased level of phosphorylated STAT3 following resveratrol treatment was ascertained by the nuclear fluorescent labeling of STAT3 (Figure 5A) as well as by Western blotting analysis (Figure 5B). Thus, resveratrol appeared to impair the inflammatory amplification loop revealed between chondrocytes and macrophages in coculture.

## 4. Discussion

The effect of resveratrol on chondrocytes has been reported previously. Indeed, Shakibaei *et al.* [20] reported that curcumin synergizes with resveratrol to stimulate the MAPK signaling pathway in human articular chondrocytes *in vitro*. In addition, Liu *et al.* [21] showed a protective effect of resveratrol in advanced glycation end product–stimulated chondrocytes. On the other hand, Baker *et al.*, [22] reported that pravastatin suppresses matrix metalloproteinase expression and activity in human articular chondrocytes stimulated by IL-1β. So *et al.* [23] published the protective effects of ginsenoside Rg3 on human osteoarthritic chondrocytes. More recently, anti-inflammatory effects of 5-HT3 receptor antagonists in IL-1β-stimulated primary human chondrocytes have been reported [24].

The novelty of the present work is the use of chondrocyte-macrophage coculture, which mimics the conditions of the biological situation in the synovial joint. This approach enabled the demonstration that in chondrocytes, the activation of NF-κB by IL-1β-induced secretion of IL-6 then triggers STAT3 activation in macrophages. In addition, STAT3 was able to positively regulate IL-6 secretion, as confirmed by the doubling level of IL-6 in cocultures compared to macrophage monocultures. These findings confirmed the usefulness of our coculture model in the elucidation of the inflammatory arthritis-linked process. We also demonstrated the presence of an inflammatory amplification loop induced by IL-1β.

**Figure 6:**
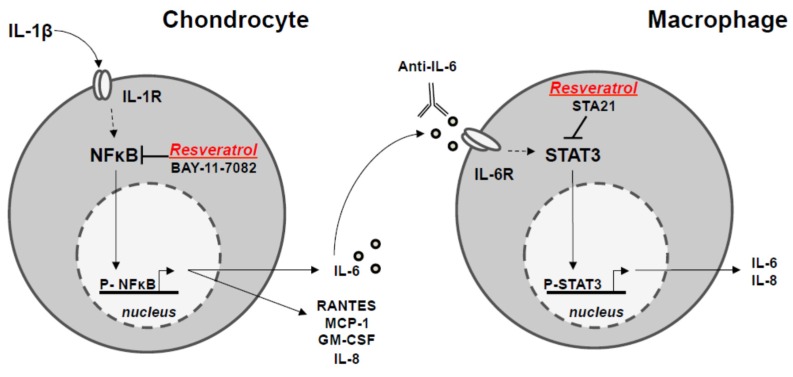
Proposed mechanism of anti-inflammatory properties of resveratrol revealed in the cross-talk between chondrocyte and macrophage in the chondrocyte/macrophage coculture model.

Concerning resveratrol, this natural compound displays strong inhibitory effects on pro-inflammatory marker secretion. The decrease of IL-6 secretion by resveratrol was dependent on NF-κB inhibition in chondrocytes. Such a reduced IL-6 level downregulates STAT3 activation in macrophages, leading to the impairment of the running inflammatory amplification loop (Figure 6). 

Our results put forward the understanding of the anti-inflammatory potential of resveratrol and open new perspectives in the prevention and possibly the treatment of osteoarthritis, based on the use of phytophenols [25,26].

After the stimulation of chondrocytes by IL-1β through the plasma membrane IL-1β receptor (IL-1β R), resveratrol leads to the inactivation of the NF-κB transcription factor by phosphorylation (p-NF-κB) of the inactive form, thus limiting the secretion of IL-6 and other cytokines (GM-CF, IL-8, MCP-1 and Rantes). Chondrocyte-secreting IL-6 can cross-talk with macrophages through the plasma membrane IL-6 receptor (IL-6R), which triggers activation of the STAT3 transcription factor by phosphorylation (p-STAT3 inactive form) and will limit IL-6 secretion. This pathway is also inhibited by resveratrol. IL-1R, IL-6R: receptors of IL-1 and IL-6.
